# Biomechanical Alterations during Sit-to-Stand Transfer Are Caused by a Synergy between Knee Osteoarthritis and Obesity

**DOI:** 10.1155/2018/3519498

**Published:** 2018-12-09

**Authors:** Loek Verlaan, Ramon J. Boekesteijn, Pieter W. Oomen, Wai-Yan Liu, Marloes J. M. Peters, M. Adhiambo Witlox, Pieter J. Emans, Lodewijk W. van Rhijn, Kenneth Meijer

**Affiliations:** ^1^Department of Orthopaedic Surgery, CAPHRI School for Public Health and Primary Care, Maastricht University Medical Center, P.O. Box 5800, 6202 AZ Maastricht, Netherlands; ^2^Department of Nutrition and Movement Sciences, NUTRIM School of Nutrition and Translational Research in Metabolism, Maastricht University Medical Center, P.O. Box 616, 6200 MD Maastricht, Netherlands

## Abstract

Osteoarthritis is one of the major causes of immobility and its current prevalence in elderly (>60 years) is 18% in women and 9.6% in men. Patients with osteoarthritis display altered movement patterns to avoid pain and overcome movement limitations in activities of daily life, such as sit-to-stand transfers. Currently, there is a lack of evidence that distinguishes effects of knee osteoarthritis on sit-to-stand performance in patients with and without obesity. The purpose of this study was therefore to investigate differences in knee and hip kinetics during sit-to-stand movement between healthy controls and lean and obese knee osteoarthritis patients. Fifty-five subjects were included in this study, distributed over three groups: healthy controls (n=22), lean knee osteoarthritis (n=14), and obese knee OA patients (n=19). All subjects were instructed to perform sit-to-stand transfers at self-selected, comfortable speed. A three-dimensional movement analysis was performed to investigate compensatory mechanisms and knee and hip kinetics during sit-to-stand movement. No difference in sit-to-stand speed was found between lean knee OA patients and healthy controls. Obese knee osteoarthritis patients, however, have reduced hip and knee range of motion, which is associated with reduced peak hip and knee moments. Reduced vertical ground reaction force in terms of body weight and increased medial ground reaction forces indicates use of compensatory mechanisms to unload the affected knee in the obese knee osteoarthritis patients. We believe that an interplay between obesity and knee osteoarthritis leads to altered biomechanics during sit-to-stand movement, rather than knee osteoarthritis alone. From this perspective, obesity might be an important target to restore healthy sit-to-stand biomechanics in obese knee OA patients.

## 1. Introduction

Osteoarthritis (OA) is one of the world's leading causes of immobility and is defined by degeneration of subchondral bone and articular cartilage in joint spaces [1]. Most commonly, OA affects weight-bearing joints such as the knee, which leads to severe alterations in biomechanics during activities of daily life [2]. According to the World Health Organisation, the prevalence of OA is 18% in elderly women, whereas this is 9.6 % in elderly men [3]. Following the current rise in obesity and concomitant increase in life expectancy, prevalence of OA is expected to increase [4]. This poses OA as an increasing future health problem. Besides age and weight, further risk factors for OA include female gender, genetics, poor diet, joint overuse, trauma, muscle weakness, physical inactivity, and poor habitual movement patterns [5, 6]. While the exact pathophysiology of OA remains to be elucidated, it is currently believed that altered joint loading and cartilage metabolism are both key factors in cartilage degradation and subsequent OA development [7].

The clinical presentation of knee OA is characterized by pain, limitation of movement, tenderness, and local inflammation [8]. Those problems often manifest at the medial tibiofemoral compartment, as a result of varus malalignment [9]. To avoid pain and overcome movement limitations, knee OA patients adopt compensatory strategies in their daily routine. In previous studies, such alterations in movement patterns have already been described in patients with knee OA during activities of daily life, including gait [10–12], stair climbing [13–15], and sit-to-stand (STS) tasks [16–21]. The ability to perform those activities effectively is essential with respect to independency and participation in society.

In this study, we will specifically investigate STS movement, which is characterized by the transition from a wide base of support (BoS), provided by the feet, thighs, and buttocks, to a small BoS, provided by the feet alone. Moreover, high knee and hip extensor moments are required to lift the centre of mass (CoM) against gravity [20]. Especially in certain pathologies, such as knee OA, where pain, joint stiffness, and loss of quadriceps strength are present, performing STS may be challenging.

From previous research it is known that knee OA patients show increased weight-bearing asymmetry [17, 18, 21], less flexion of the affected knee [16, 18], increased trunk lean towards the unaffected side [17], and more flexion of the trunk [16, 17, 20] during STS movement. Furthermore, lower knee extension moments are observed [19, 20], which is associated with lower quadriceps strength [18]. The observed movement alterations are also linked with earlier and increased activation of the biceps femoris [16, 19]. Overall, those movement alterations lead to an increased time to perform STS movement, indicating a decrease in performance [20, 22]. However, performing STS at a slower speed may also be a deliberate strategy to reduce accelerations and minimize both joint forces and joint pain [23]. As a result of compensatory movement patterns, in particular asymmetrical loading strategies, the contralateral joint may become more prone to developing OA [24]. This underlines the importance of proper quantification of biomechanics during STS, which may lead to the prevention of further disease progression.

Although there are quite some studies that have investigated the effects of knee OA on STS movements, most of those studies fail to distinguish between effects of OA itself and effects of high body mass index (BMI), which is closely associated with OA. As obesity itself may modulate movement patterns during STS, it should not be neglected in biomechanical analyses [25]. Furthermore, obesity is one of that factors that may contribute to OA progression [26]. Therefore, we investigated the differences in knee and hip kinetics during STS movement between healthy controls and lean and obese knee osteoarthritis patients. In addition, we investigated different kinds of employed compensatory strategies to perform STS movements. We hypothesize that the combination of obesity and knee osteoarthritis is responsible for altered knee and hip kinetics and an increase in time during STS transfer, rather than knee osteoarthritis alone. To overcome joint pain, we expect that obese knee OA patients will increase loading of the unaffected leg, which increases their time to rise from a chair.

## 2. Materials and Methods

### 2.1. Study Population

In this case-control study three groups were studied: healthy controls (BMI = 20-25 kg/m^2^), lean knee OA patients (BMI = 20-25 kg/m^2^), and obese knee OA patients (BMI = 30-40 kg/m^2^). Subjects having a Kellgren-Lawrence (KL) score between 1 and 3 at the medial tibiofemoral site were included in the OA groups [27]. The specific focus on medial knee OA is related to its prevalence and relation with increased external knee adduction moments during locomotion [28]. Only women aged between 50 and 65 years were included, as knee OA prevalence is the highest in this group. The upper age limit was adopted to prevent inclusion of participants that are at high risk of having comorbidities. Recruitment of knee OA patients occurred via the ‘Artrose Kliniek' at the Maastricht University Medical Center (MUMC+). Healthy controls were recruited by the Department of Nutrition and Movement Sciences, the Department of Physical Therapy (MUMC+), and local physical therapy clinics in Maastricht.

Exclusion criteria were any inflammatory arthritis, trauma, OA at any other joint, and moderate to severe OA in the ipsilateral patellofemoral OA and/or lateral tibiofemoral OA, anterior cruciate ligament injury, medial and collateral ligament injury, and psychiatric illness according to the Diagnostic and Statistical Manual of Mental Disorders classification criteria for psychiatric illnesses (patients were excluded when diagnoses were present in their medical files). Healthy women were nonobese, did not meet the exclusion criteria, and did not have knee OA according to the American College of Rheumatology classification criteria [29]. Absence of knee OA in the control group was also ensured by Magnetic Resonance Imaging (MRI).

All subjects were informed on the purpose of the study and gave informed consent before participating in this study. This study was ethically approved by the METC aZM/UM.

### 2.2. Radiographic Analysis

Radiographic imaging was used to evaluate knee cartilage health and knee OA status. Presence of knee OA was assessed from X-ray images by the KL knee score [27]. The X-ray images were evaluated double blind by two independent orthopaedic surgeons.

To more accurately assess cartilage health in all study groups, MRI was performed using a 3T Philips Intera Scanner* (Philips Medical Systems, Best, The Netherlands)*. Scanning sequences included fat saturated proton density-weighted turbo spin echo and fat saturated T2 weighted sequences. Cartilage health in the knee was evaluated based on the MRI Osteoarthritis Knee Score (MOAKS) [30]. In this semiquantitative scoring system, the knee is subdivided into 14 regions which are scored on seven features. The 14 subregions include different sites at the patella, femur, and tibia: the medial and lateral patella; the medial and lateral trochlea, the medial and lateral central femur, and the medial and lateral posterior femur; the medial and lateral anterior tibia, the medial and lateral central tibia, and the medial and lateral posterior tibia. In the present study, only the articular cartilage feature of the MOAKS was scored. The articular cartilage score provides separate scores for the size and depth of cartilage damage in each of the subregions. The size of any cartilage loss (partial and full-thickness loss) as well as the size of full-thickness cartilage loss was scored as a % of surface area as related to the size of each individual region as either 0 (none), 1 (<10% of region of cartilage surface area), 2 (10-75% of region of cartilage surface area), 3 (>75% of region of cartilage surface area), or N (no score possible). To correct for cases where scoring was not possible, the total MOAKS score was divided by the number of items scored.

### 2.3. Instrumentation

Movement analysis was performed with an eight-camera, three-dimensional (3D) motion capture system* (Vicon, MX3, Oxford Metric, UK)* together with Nexus software. Kinetic data were obtained by one force platform* (9281A, Kistler instruments AG, Winethur, Switzerland)* which measured ground reaction force in order to calculate joint torques and forces. Sixteen reflective markers were placed on the lower extremities according the Vicon Plug in Gait model in order to use the 3D motion capture system. In the obese knee OA group, however, it was sometimes necessary to deviate from the model, as the abdominal fat depot limited visibility of the spina iliaca anterior superior. In those cases, markers were placed more lateral and/or dorsal, according to the Vicon Plug in Gait Marker Placement Manual.

### 2.4. Sit-to-Stand Task

Subjects were asked to rise from a chair on a self-selected, comfortable speed. The chair had no arm and backrests and height was adjusted to knee and hip angles of 90 degrees. Use of the arms was prohibited, which was ensured by positioning each hand on the contralateral shoulder. Further, trials were performed barefoot and feet were placed parallel and in line with the shoulders. The dominant (control group) or affected (knee OA groups) leg was placed on the force platform. Leg dominance was assessed by asking the subject which leg would be used to kick a ball. After completion of the STS transfer, subjects were asked to sit again from the obtained standing position. Two test trials were performed to get familiar with the movement. Measurements were repeated seven times with 10 seconds of resting intervals.

### 2.5. Data Analysis

Data were processed via MATLAB to generate the variables of study. Parameters of interest were total time, subphase duration, ankle/knee/hip ROM in the sagittal plane, ankle/knee/hip extension moments, knee adduction moments, and the vectors of the ground reaction forces: anterior-posterior (GRFy), vertical (GRFz), and mediolateral (GRFx). Joint moments and GRFz were corrected for body weight (BW). Trials were normalized to 100% of the STS task with intervals of 0.5%. The start of the trial was defined by the first moment the GRFz exceeded 20% of the maximal GRFz, with a threshold of 40 N. End of the trial was defined by the moment when the GRFz was lower than 20% of the maximal GRFz. Trials were subdivided into three phases based on joint kinematic events. Those phases included the leaning phase (start, maximal hip flexion), momentum phase (maximal hip flexion, maximum ankle dorsiflexion), and extension phase (maximum ankle dorsiflexion, end of trial) [31].

### 2.6. Statistical Analysis

Normality of data was tested with the Shapiro-Wilk test. Averages were calculated over the different trials for the following parameters: STS time, subphase duration, GRF (all vectors), ankle/knee/hip sagittal ROM, ankle/knee/hip sagittal moments, and frontal knee moments. Reliability of the kinetic data for the knee and hip was tested using the intraclass correlation (ICC) [32]. Group differences for STS parameters were analysed with one-way ANOVA using LSD post hoc analysis and nonparametric Kruskal Wallis tests using pairwise comparisons with Bonferroni adjustments. Data are presented as mean ± standard deviation (SD). The level of agreement for the Kellgren-Lawrence scoring was tested with Cohen's kappa. The relations between knee OA severity and BMI and knee OA severity and time to perform the STS were tested with Pearson correlations. Significance level was set at *α*<0.05. All statistical analysis was performed with IBM SPSS statistics 24.

## 3. Results

### 3.1. Subject Characteristics

Fifty-five subjects were included in this study (Table 1). No significant differences in age were found between the three groups. The obese group had a higher body mass and was shorter than both the controls and lean knee OA group. Consequently, BMI was significantly higher in the obese knee OA group. Radiographic analysis indicated that both knee OA groups show average KL scores between two and three, confirming the presence of knee OA. Concordance between both orthopaedic surgeons was substantial (*κ*=0.639). The sum of MOAKS, corrected for the number of items scored, further demonstrated absence of meaningful knee OA in the control group, as it differed significantly from both the lean knee OA group (p=0.027) and obese knee OA group (p<0.001). Furthermore there was no significant difference in MOAKS between the two OA groups.

### 3.2. STS Time

No significant differences between groups were observed in the duration of the STS task. (Table 2). The relative contribution of all subphases was not significantly different between groups. In all groups, duration of the extension phase was relatively the longest (64.1%–68.7%), while duration of the leaning phase (17.8%-21.2%) and momentum phase (12.1%-16.0%) contributed less to STS duration. However, time to perform the first trial was significantly higher than the last trial in the knee OA group (p=0.025), pointing towards a learning effect.

### 3.3. Kinetics and Kinematics

**Figure 1:**
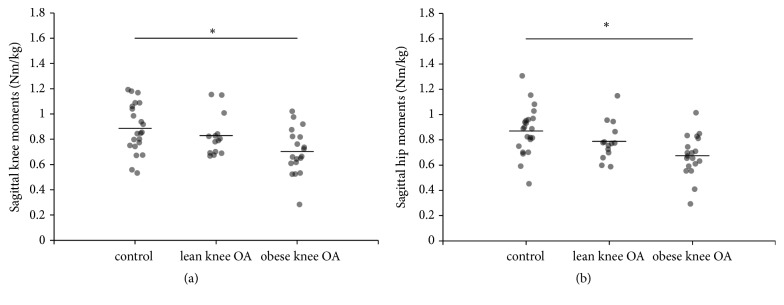
Sagittal joint moment during STS transfer for the three different groups for knee (a) and hip (b). The horizontal lines indicate the mean group values.**∗**p<0.05.

Kinetic data for the knee and hip in the sagittal plane showed high repeatability with an ICC of 0.970 and 0.917, respectively. Knee ROM in the sagittal plane was significantly lower in the obese knee OA group compared to both healthy controls (p=0.007) and lean knee OA patients (p=0.009). Similarly, hip ROM was significantly lower in the obese knee OA group, compared to healthy controls (p=0.023) and lean knee OA patients (p=0.009). The reductions in knee and hip ROM corresponded with lower maximal knee (p=0.002) and hip extension moments (p=0.001) in the obese knee OA group compared to the control group (Figure 1). For the ankle, no significant differences in ROM and joint moments were found between groups. Maximal knee adduction moments did not differ between groups.

### 3.4. Ground Reaction Force

The maximum of GRFz, after correction for bodyweight, was lower in the obese knee OA group, when compared to healthy controls (p=0.001). No differences in GRFz were found between the lean knee OA group and the controls. GRFx was higher in the obese knee OA group compared to both lean knee OA (p=0.045) and controls (p=0.003). Similarly, GRFy was higher in the obese knee OA group compared to both the lean knee OA patients (p=0.005) and healthy controls (p=0.01).

### 3.5. Correlations

A significant correlation between knee OA severity, defined by the sum of the MOAKS score divided by the number of items scored, and time to perform the STS transfer was found (r=0.338; p=0.02).

## 4. Discussion

The aim of the current study was to investigate differences in knee and hip kinetics during STS movement between healthy controls and lean and obese knee OA patients. Second, use of compensatory strategies was investigated in the different study groups. We were able to show reduced knee and hip ROM, accompanied by reduced peak hip and knee moments in the obese knee OA group. In addition, GRFz corrected for bodyweight was lower in the obese knee OA group compared to the control group. Obese subjects also showed a greater GRFx than both other groups, indicating the use of compensatory mechanisms to unload the affected knee. Total time to perform STS was not different between groups. Furthermore, none of the investigated STS parameters was different between the lean knee OA group and controls.

In previous research it has been shown that knee OA patients show alterations in STS movement [16–22]. To our knowledge, none of those studies distinguishes between effects of high body mass and effects of knee OA in this impairment. Strikingly, lean subjects with knee OA did not show any signs of movement alterations during STS. Contrarily, we showed that only obese knee OA patients have different STS movement patterns. In line with others, we were able to show reductions in ROM for the knee and hip in the sagittal plane for obese knee OA patients [16, 18]. Bouchouras et al. speculate that this reduction may be caused by greater agonist-antagonist coactivation. By early recruitment of the biceps femoris compared to the vastus lateralis, the affected joint would be protected from extreme excursions and pain could be avoided. Furthermore, cocontraction could increase joint stiffness and limit joint range of motion [16]. Unfortunately, electromyographic data in this study was not included. Therefore, it was not possible to substantiate mechanisms of altered cocontraction and coactivation in the obese knee OA population. Nevertheless, pain avoidance is considered as a reasonable explanation for the reduced ROM. Considering the correlation between BMI and knee OA severity, it seems plausible that obese knee OA patients experience more pain than lean knee OA patients and thus avoid extreme joint excursions. However, future studies should first establish the relation between pain and obesity in knee OA patients.

Adequate hip and knee extension forces are also essential for efficient STS performance [20]. During STS movement, obese knee OA patients display lower peak extension moments for both knee and hip. According to Sibella et al. obese subjects without knee OA tend to unload their lower back by transferring the load to their knees [25]. We provide evidence that this statement is not applicable to obese subjects with knee OA, as knee extension moments are decreased in obese knee OA patients. Compensatory strategies, such as weight-bearing asymmetry and lateral trunk lean, are proposed to reduce sagittal joint moments at the affected side of knee OA patients with pain alleviation as primary goal [17, 18]. Although we did not measure trunk biomechanics, our results show a decreased GRFz after correction for body weight and an increased GRFx in the obese knee OA group only. Apparently, obese knee OA subjects tend to unload their affected leg by displacing the body towards the unaffected side, whereas lean knee OA patients do not show this adaptation. We therefore suggest that the compensatory strategies previously reported in literature, including weight-bearing asymmetry, only occur when both obesity and knee OA are present [17, 18].

Generally, STS performance is quantified by the total time to perform the task. Although we did expect to find differences in STS duration, total time was not significantly different between groups, which is in contrast with studies of Su et al. and Turcot et al. [17, 22]. In their studies, the increase of time was attributed to use of compensatory strategies. Differences in knee OA severity may possibly underlie this discrepancy in findings, as our results show a positive correlation between knee OA severity and STS time. The current study included only mild to moderate medial knee OA patients (KL score = 2-3) with unilateral involvement, whereas the study of Turcot et al. included obese end-stage knee OA patients (KL score = 4) and Su et al. included both unilateral and bilateral knee OA patients with unknown BMI. We therefore conclude that there is no increase in STS time in both lean and obese patients with mild to moderate OA. It might have occurred that knee OA severity was not high enough to find a significant increase in time in the obese knee OA group.

In short, alterations in lower limb biomechanics seem to be only apparent in presence of both obesity and knee OA during STS movement. Within this group, compensatory mechanisms might be necessary to avoid pain and to preserve the ability to perform the task. Although our current study design allowed distinguishing between effects of knee OA and obesity, there were some limitations. We could not explain the occurrence of compensatory mechanisms by pain avoidance, as pain was not measured in this study. Besides, no markers were placed on the trunk to investigate its role in movement adaptations. Furthermore, muscle activity was not measured. Future studies on STS movement should be performed with a similar study design that includes electromyographic data, trunk biomechanics, and pain measurements. Finally to investigate the exact influence of BMI, our suggestion would be to include a second control non-OA obese group.

## 5. Conclusion

Our study shows that the biomechanical alterations during sit-to-stand movement are the result of an interplay between high body mass and knee OA, rather than knee OA alone. The combination of obesity and knee OA leads to reduced ROM in the knee and hip of the affected leg. Similarly, peak extension moments are decreased in both joints. This might be explained by asymmetrical loading, characterized by a lower GRFz, corrected for bodyweight, and higher GRFx of the affected leg. Since only obese knee OA patients show movement alterations, losing weight could restore sit-to-stand biomechanics to a healthy pattern. Future studies should examine the differences in muscle activity and trunk biomechanics between the different study groups for more insight in employed compensatory strategies.

## Figures and Tables

**Table 1:** Patient characteristics of the three different study groups, presented as mean (± SD).

***Demographics***	***Group***		
	Control (n=22)	Lean knee OA (n=14)	Obese knee OA (n=19)
Age (years)	58.7 (4.4)	60.1 (3.5)	59.0 (5.1)
Height (m)	1.66 (0.04)	1.67 (0.05)	1.62 (0.07)^1,2^
Weight (kg)	62.9 (6.1)	66.1 (7.3)	86.4 (12.3)^1,2^
BMI (kg/m^2^)	22.5 (2.0)	23.7 (2.3)	32.4 (3.4)^1,2^
KL-score	-	2.21 (0.74)	2.38 (0.70)
MOAKS (score/items)	0.50 (0.42)	1.01 (0.69)^1^	1.22 (0.66)^1^

*Note*. BMI = body mass index, KL = Kellgren-Lawrence, MOAKS = MRI osteoarthritis knee score, and OA = osteoarthritis.

1 = significantly different from control.

2 = significantly different from lean knee OA.

**Table 2:** STS-parameters for all different groups. Data are presented as mean (±SD).

***STS-parameter***	***Group***		
	Control (n=22)	Lean knee OA (n=14)	Obese knee OA (n=19)
*STS time (s)*			
Total	0.99 (0.21)	1.11 (0.28)	1.17 (0.43)
*Sub-phase duration (%)*			
Leaning phase	17.8 (6.1)	19.2 (6.2)	21.2 (9.6)
Momentum phase	16.0 (6.0)	12.1 (4.7)	14.7 (5.1)
Extension phase	66.2 (7.0)	68.7 (5.1)	64.1 (7.8)
*Joint ROM (*°)			
Ankle (sagittal)	18.8 (6.6)	18.9 (6.1)	14.2 (6.5)
Knee (sagittal)	85.5 (11.4)	85.8 (8.2)	75.9 (10.3)^1,2^
Hip (sagittal)	79.7 (7.4)	81.7 (5.4)	73.1 (12.3)^1,2^
*Maximum joint moment (Nm/kg)*			
Ankle (sagittal)	0.32 (0.12)	0.32 (0.09)	0.29 (0.08)
Knee (sagittal)	0.89 (0.20)	0.83 (0.16)	0.70 (0.18)^1^
Knee (frontal)	0.30 (0.22)	0.26 (0.21)	0.27 (0.18)
Hip (sagittal)	0.87 (0.19)	0.79 (0.15)	0.67 (0.16)^1^
*Ground reaction force (BW)*			
GRFz max	0.62 (0.06)	0.58 (0.05)	0.54 (0.08)^1^
*Ground reaction force (N)*			
GRFz max	374.6 (48.8)	376.2 (52.9)	459.0 (89.4)^1,2^
GRFx max	33.9 (8.22)	37.1 (10.2)	45.9 (16.5)^1,2^
GRFy max	37.4 (12.2)	34.9 (12.4)	47.7 (12.6)^1,2^

*Note*. STS = sit-to-stand, ROM = range of motion, BW = bodyweight, GRFz = vertical ground reaction force, GRFx = medio-lateral ground reaction force, GRFy= anterior-posterior ground reaction force, and OA = osteoarthritis.

1 = significantly different from control.

2 = significantly different from lean knee OA.

## Data Availability

The data used to support the findings of this study are included within the article.
